# Deep learning-driven anomaly detection and feature discovery in Ce-rich (Ni–Fe–Co–Ce)O_*x*_ catalysts for oxygen evolution reaction

**DOI:** 10.1039/d6ra02168a

**Published:** 2026-05-20

**Authors:** Chih-Yang Cheng, Yi-Huan Wu, Feng-Yin Li

**Affiliations:** a Department of Chemistry, National Chung Hsing University Taichung 402 Taiwan feng64@nchu.edu.tw; b Department of Chemistry, R. O. C. Military Academy Kaohsiung Taiwan m1090008@rocma.edu.tw

## Abstract

Developing high-performance oxygen evolution reaction (OER) catalysts is vital for energy conversion. However, extracting rare, exceptional materials from massive high-throughput experimental datasets remains challenging, as conventional machine learning models often misclassify these optimal data points as noise. Here, we propose a deep learning-driven anomaly detection framework that overcomes this limitation. By integrating atomic-level descriptors with convolutional neural networks (CNNs) for similarity stabilization analysis, our model employs an iterative data-cleaning mechanism to automatically isolate and evaluate high-performing outliers. We validated this approach on a high-throughput (Ni–Fe–Co–Ce)O_*x*_ catalyst dataset. The model achieved a robust *R*^2^ score of 0.90 for inlier predictions while successfully capturing a specific optimal compositional window of Ce-rich compositions (0.3–0.6 at%) exhibiting remarkably low overpotentials. This framework offers a reliable, data-driven analytical tool, demonstrating the power of deep learning anomaly detection in accelerating the discovery and optimization of novel materials.

## Abstract 2

VisOut, a deep learning framework, autonomously identifies high-performance Ce-rich OER catalysts from noisy datasets. Isolating specific optimal compositional windows accelerates data-driven discovery of unconventional electrocatalysts.

## Graphical abstract

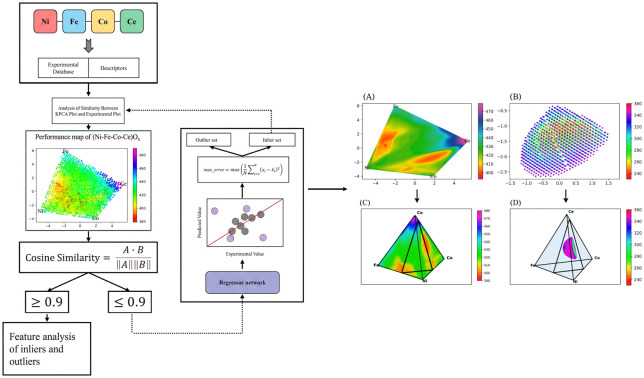

## 1 Introduction

The oxygen evolution reaction (OER) is a critical bottleneck in renewable energy technologies, such as water splitting and metal-air batteries, where efficient catalysts are essential to overcome the high overpotential (OP) and sluggish kinetics inherent to this reaction.^1–3^ Transition metal oxides, particularly those based on Ni–Fe–Co systems, have been extensively studied due to their high catalytic activity, cost-effectiveness, and scalability in industrial applications.^4–6^ Recent advancements in high-throughput experimental techniques have revealed that incorporating cerium (Ce) into Ni–Fe–Co catalysts can markedly enhance catalytic performance by exploiting the unique electronic properties of Ce and its synergistic effects with other transition metals.^7,8^ For instance, Haber *et al.* reported that an electrodeposited Ni_0_._2_Co_0_._3_Ce_0_._5_O_*x*_ composition achieves 10 mA cm^−2^ oxygen evolution current at an overpotential as low as 310 mV.^9^

High-throughput experimental techniques have emerged as transformative tools for catalyst discovery, enabling the rapid generation of large datasets that capture both compositional and electrochemical properties.^10–12^ However, the rapid data acquisition intrinsic to high-throughput methods often introduces noise, measurement errors, and anomalous data points.^13^ Historically, conventional machine learning (ML) applications in materials science have predominantly focused on “data fitting” optimizing models to accurately predict the bulk of the dataset.^14–17^ Consequently, exceptionally high-performing data points are frequently misclassified as statistical noise or outliers and subsequently discarded.^18^ This fundamental limitation prevents traditional ML models from achieving true materials discovery.

In this study, we present a novel deep learning-driven anomaly detection framework designed specifically to overcome this limitation.^18^ To rigorously validate the capability of our proposed framework, we adopt the comprehensive high-throughput (Ni–Fe–Co–Ce)O_*x*_ dataset reported by Haber *et al.*^9^ as our established “ground truth.” Unlike conventional approaches that filter out anomalies to artificially boost model accuracy, our framework purposefully isolates and analyzes these outliers to uncover potential new catalytic features through an automated similarity stabilization analysis. The primary motivation of this research is to demonstrate how an automated machine learning pipeline operating entirely without *a priori* chemical intuition or human intervention can independently rediscover the rare, high-performance compositional rules that human experts previously uncovered through painstaking empirical analysis.^19^ By incorporating advanced techniques such as kernel principal component analysis (KPCA) for dimensionality reduction and convolutional neural networks (CNNs) for compositional similarity analysis, our model autonomously converges upon the optimal Ce-rich feature space. This successful “rediscovery” serves as a robust proof of concept, powerfully validating that our deep learning approach can reliably unearth novel, hidden catalytic behaviors from massive datasets.^20,21^ Ultimately, this study shifts the paradigm of ML in catalyst optimization from a mere data-fitting tool into an independent, data-driven engine for accelerating the discovery of unconventional, high-efficiency materials.

## 2 Methods

**Fig. 1:**
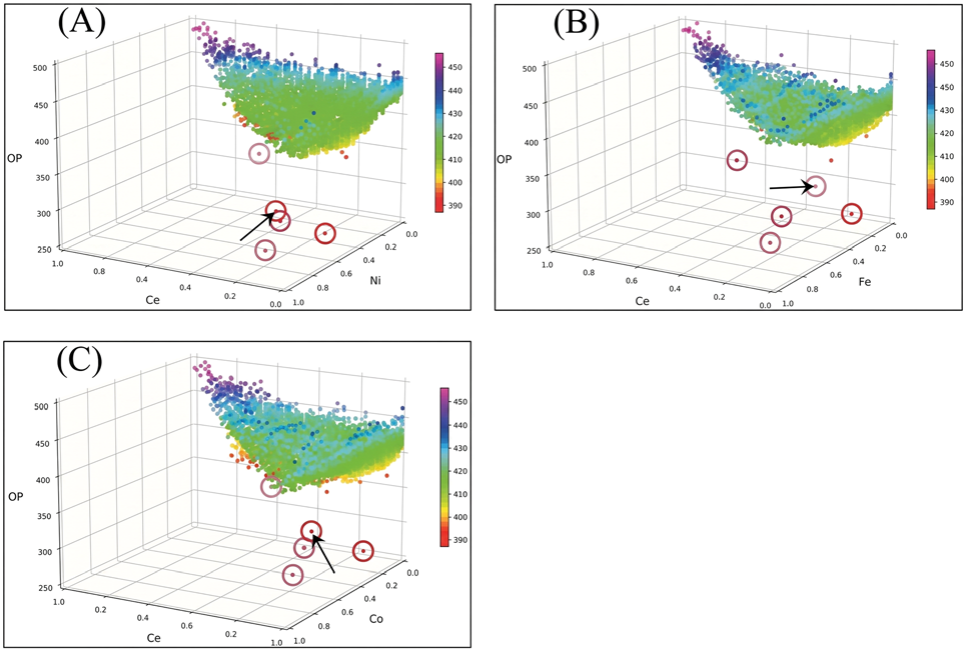
Compositional analysis of OP values in Ce-rich catalysts. The distribution of OP values is plotted as a function of Ce atomic composition against each of the other three metal compositions: (A) Ni, (B) Fe, and (C) Co. Red circles highlight five data points with exceptionally low OP values, indicating potentially advantageous compositional regions. The arrow marks the data point predicted to be erroneous.

We aim to design a suitable deep learning model to uncover potential underlying features in high throughput experimentation that traditional methods might overlook. As suggested by Haber *et al.*,^9^ we first examine how Ce composition influences catalytic performance by plotting the OP values as a function of the Ce compositional ratio against one of the other three metals, as shown in Fig. 1. Most OP values fall within the range of 400–450 mV. However, several exceptionally low OP values, scattered between 200 and 350 mV, appear in Ce-rich compositions. This observation suggests that Ce plays a significant role in enhancing catalytic activity. Rather than attributing these data to measurement errors, the five highlighted points in Fig. 1 may represent novel compositional structures that provide new insights into catalyst design. Accordingly, our model seeks to identify these unique features in high throughput experimentation that conventional approaches may fail to capture. Notably, these exceptional data points pose a serious challenge for grouping based on OP values. To address this issue, we designed deep learning models that incorporate a data filtering process to handle anomalous data points in experimental measurements, thereby enhancing predictive accuracy.^13^

### 2.1 Details of workflow

**Fig. 2:**
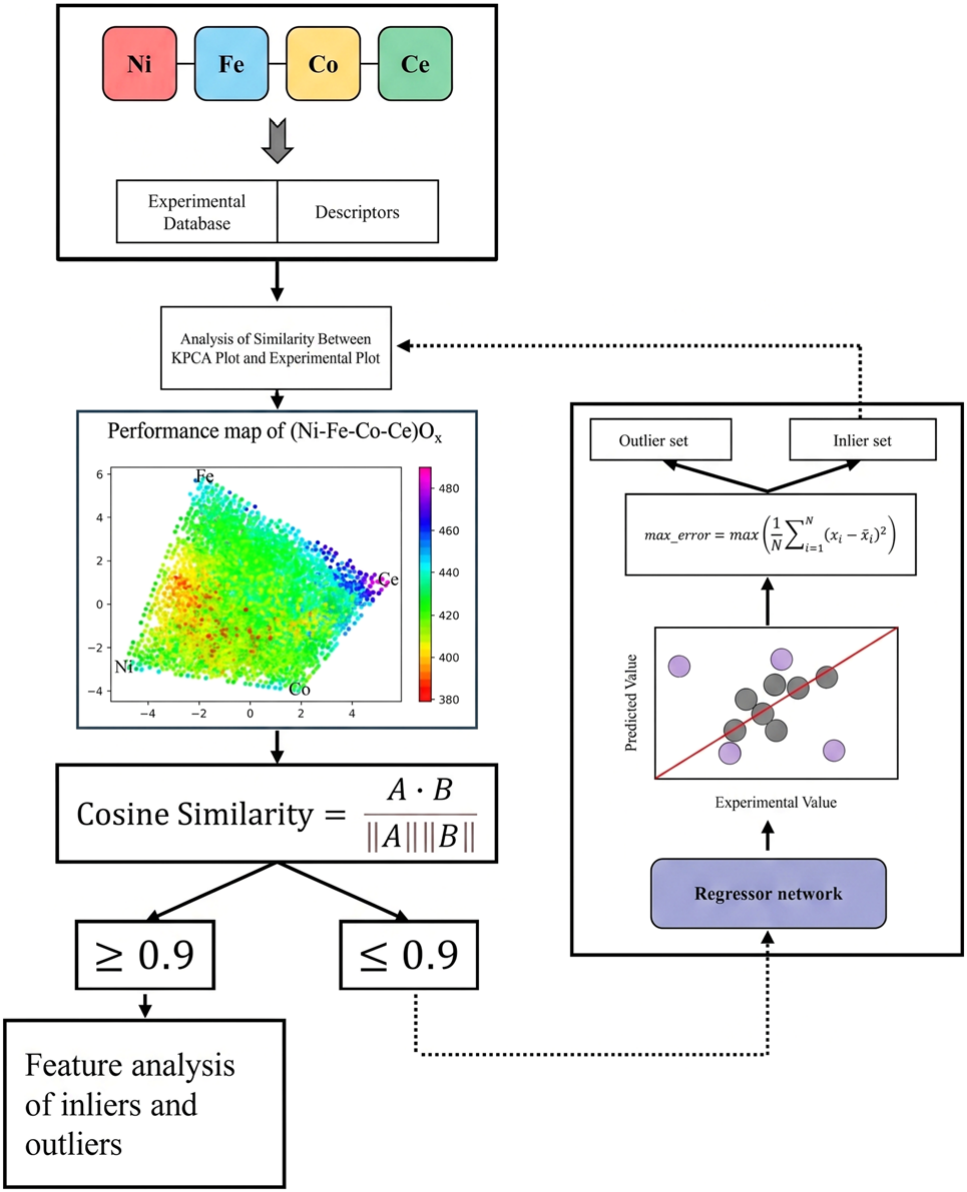
Workflow diagram for overpotential prediction in Ce-rich catalysts. The workflow illustrates a regression-based framework designed to predict catalytic overpotential in Ce-rich multimetallic oxides. Experimental metal composition data are integrated with quantitative descriptors to construct the regression network, enabling precise overpotential prediction and identification of regions associated with maximal prediction uncertainty within Ce-enriched catalyst systems.

To ensure that regression training is based on the most physically consistent data and to enhance the model learning rate, we developed VisOut™ (Visual Similarity of Outlier Trimming). This approach combines iterative error analysis with image-based similarity tracking, allowing compositional outliers to be progressively removed while preserving the core chemical patterns. The VisOut™ workflow functions as a decision loop (Fig. 2) that processes experimental data in an iterative manner.

For input preparation, descriptive parameters were appended to each experimental data entry, *i.e.*, the metal composition together with its OP value was treated as a single data point, and all data points were concatenated into an input vector. The selected descriptive parameters are provided in the SI Materials. Although atomic descriptors remain constant, their compositional projections vary across samples, embedding chemical knowledge into the learning space through structure property correlations that can be more readily captured. This approach is consistent with recent advances in materials informatics, where similar strategies have been shown to significantly enhance prediction performance and model interpretability.

For example, Liu *et al.* demonstrated that enriching input features with elemental descriptors improved regression accuracy in perovskite property prediction tasks.^22^ This high-dimensional feature space was employed consistently throughout the workflow.

To describe the details of VisOut™, we focus on the filtering endpoint identifier: the Visual Similarity Check. This process consists of two components: (i) a Kernel Principal Component Analysis (KPCA) visualizer and (ii) a graphical comparator using cosine similarity. To visualize the underlying features of the dataset, we employed KPCA, a non-linear extension of PCA, implemented in the scikit-learn package to construct a composition map.^23^ While the learning map can be distorted by discrepant data, the experimental map more faithfully represents the dataset's inherent characteristics, serving as a convenient reference for the composition map of the regular data points, which constitute the majority of the dataset. To quantify the difference between the learning and experimental composition maps, we used cosine similarity, defined as:^24^1
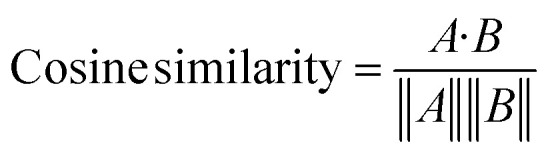
Here, *A* represents the deep feature vector extracted from the experimental map, and *B* denotes the corresponding vector from the current KPCA-based learning map. These high-dimensional vectors were obtained using VGG16, a convolutional neural network pre-trained on ImageNet.^25^ In this framework, VGG16 is deliberately utilized as an “off-the-shelf feature extractor” functioning within a content-based image retrieval (CBIR) paradigm.

Although originally developed for natural image recognition, the shallow layers of pre-trained CNNs are highly adept at capturing fundamental visual elements such as edges, shapes, and color gradients. These low-level visual representations correspond well to the data clusters and gradient patterns in our KPCA heatmaps. The validity of this zero-shot feature extraction approach is well-supported by recent literature, which demonstrates that representations extracted from pre-trained CNNs can be successfully transferred to the clustering, comparison, and classification of scientific figures, heatmaps, and biomedical compound images.^26^ A similarity threshold of 0.9 was selected for the trimming endpoint because the experimental map still contains contributions from outlier data points, and the similarity score typically plateaued around 0.9 during the actual run.

One may ask why image-based cosine similarity was preferred over conventional statistical distance measures such as KL divergence. Traditional statistical metrics primarily evaluate point-by-point probability distribution differences, and are therefore insensitive to the geometric topology and boundary structure of high-dimensional data once projected into a low-dimensional KPCA map.

The KPCA composition maps encode overpotential gradients as spatial color patterns and cluster shapes structural features that are captured naturally by the shallow convolutional layers of VGG16, which are specifically optimized to detect edges, local contrast, and shape boundaries. This allows the similarity check to operate analogously to a chemist visually comparing two composition-performance landscape images, capturing holistic topological changes that a scalar statistical distance would fail to register. The use of a pre-trained CNN as an off-the-shelf visual feature extractor in this zero-shot content-based image retrieval paradigm is well supported by recent literature.^25,26^

To empirically validate this visual similarity metric and ensure the convergence is not a colormap-induced artifact, we calculated the direct pixel-level Pearson correlation between the KPCA map and experimental plot, and cross-validated the results using an alternative perceptually uniform colormap (plasma) (SI Fig. S1). Both approaches confirmed a statistically significant spatial correspondence.

### 2.2 OP predictor: feature engineering of OP values

The OP values of the input dataset were predicted using a deep learning regression network. The input layer comprises 60 features, including 4 elemental compositions and 56 molecular descriptors; the specific definitions and calculation methods for these 14 descriptors per element, along with their computed numerical values for each of the four building-block elements ([Ni], [Fe], [Co], [Ce]), are detailed in SI Table S1. The deep learning model utilizes three fully connected hidden layers (256, 128, and 64 neurons) with a Leaky ReLU activation function. A comprehensive summary of the network architecture, including specific training hyperparameters such as learning rate, batch size, and the dropout rate used to mitigate overfitting, is provided in SI Table S2.

To ensure robust evaluation, the dataset was randomly divided into training and testing subsets in an 8 : 2 ratio. After training the OP predictor, the absolute prediction error for each data point *i* was calculated as:

2 *δ*_*i*_ = |*y*_*i*_ − *ŷ*_*i*_|

where *y*_*i*_ is the experimental OP value and *ŷ*_*i*_ is the model prediction. The data point with the largest deviation *δ*_max_ is identified and transferred to a separate outlier set, while the remaining points are collected as the input for the filtering endpoint identifier in the subsequent iteration.

### 2.3 Feature analyzer

The artificial neural network (ANN), implemented in Python, was selected for the anomaly detection process due to its speed, simplicity, and robustness. The ANN architecture consisted of hidden layers arranged in a 256-128-64 configuration, with ReLU employed as the activation function.^27^

## 3 Results and discussion

### 3.1 Data cleaning process and its impact on learning

**Fig. 3:**
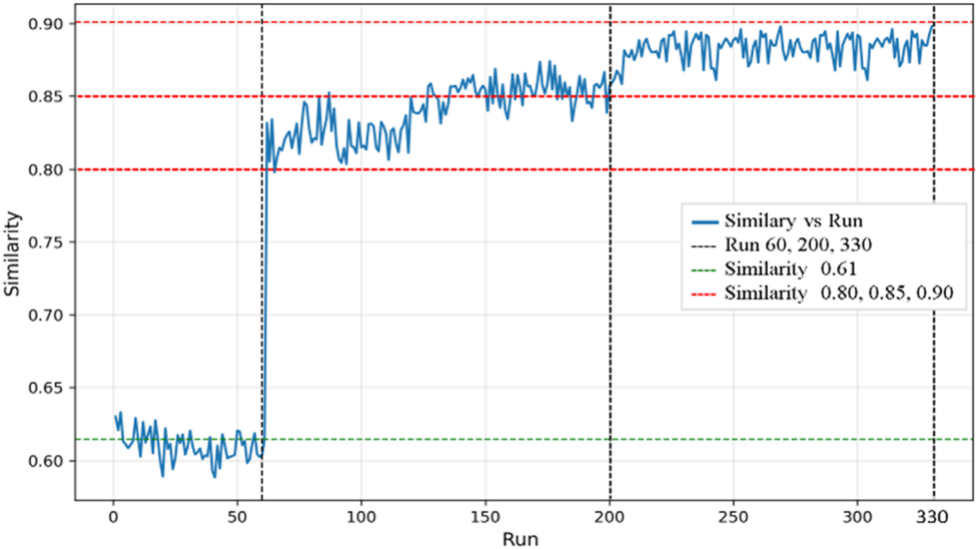
Compositional similarity analysis during iterative outlier identification. The *x*-axis represents the number of iterations in which maximum error data points were removed, and the *y*-axis shows similarity scores between experimental images and KPCA generated maps obtained using CNN based scoring. Key transitions are observed at iteration 60 (similarity increases from 0.61 to 0.84), between iterations 60–200 (fluctuations around 0.80 ± 0.03), and between iterations 200–330 (stabilization at 0.90 ± 0.02).

**Fig. 4:**
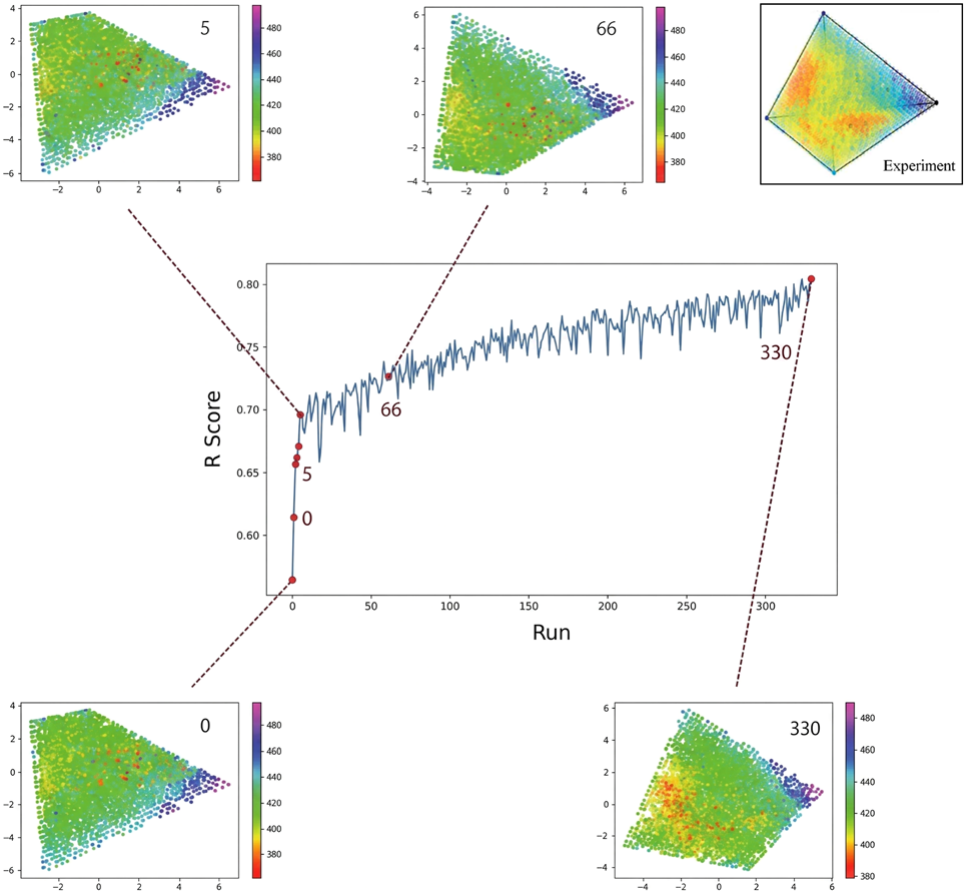
Evolution of predicted OP composition maps during the data cleaning process. The *x*-axis represents the number of outliers removed, and the *y*-axis shows the *R*^2^ training score. The inset maps illustrate the progressive transformation of predicted OP composition maps as outliers were iteratively removed.

The data cleaning process was critical for improving the predictive accuracy of Ce-rich OER catalysts. By iteratively removing outliers, the model achieved a substantial enhancement in performance. Initially, the predicted OP composition map exhibited a trapezoidal shape with a low *R*^2^ training score of 0.56, indicating poor predictive quality. After the removal of the first five outliers, the *R*^2^ training score improved to 0.69. At the 66th removal, the map transformed into a tetrahedron-like structure, although it still differed from the experimental map, with the *R*^2^ training score reaching 0.73. The process continued until 330 outliers had been removed, at which point the *R*^2^ training score reached 0.80. The stopping criterion was determined using similarity stabilization analysis (Fig. 3), which showed minimal changes in similarity scores between 250 and 330 iterations. These results confirm the effectiveness of the data cleaning process in isolating outliers and refining the inlier dataset to enhance model performance (Fig. 4).^28^

### 3.2 OP distributions of inliers and outliers

**Fig. 5:**
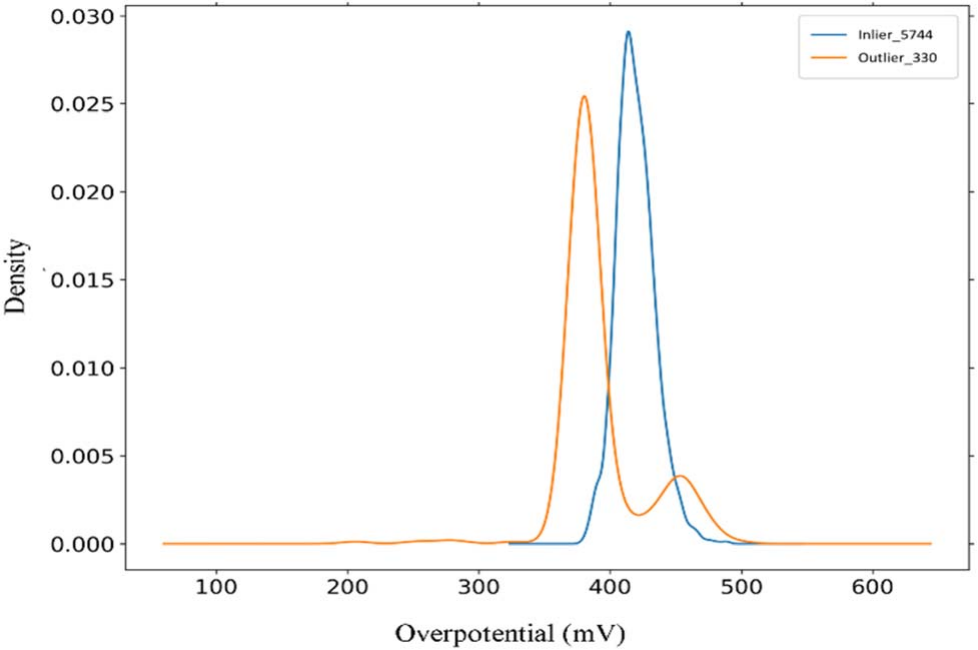
OP distributions of inliers and outliers. Inliers exhibit a unimodal distribution concentrated in the range of 380–480 mV, whereas outliers display a bimodal distribution with peaks at 386 mV (low OP mode) and 455 mV (high OP mode). The bimodal distribution of outliers highlights the presence of a novel catalytic feature in the low OP mode, which aligns with Ce rich compositions. The high OP mode, however, is less relevant to the focus of this study.

The OP distributions of inliers and outliers after data cleaning exhibit distinct patterns (Fig. 5). The inliers follow a unimodal distribution concentrated in the range of 380–480 mV, representing the primary feature space of the dataset. By contrast, the outliers display a bimodal distribution with peaks at 386 mV (low OP mode) and 455 mV (high OP mode). The low OP mode is particularly significant, as it correlates with specific metal composition ratios that are critical for efficient OER performance. The high OP mode, although present, is less relevant to the focus of this study on efficient OER catalysts. These results suggest that the low OP values observed among the outliers may represent a novel catalytic feature.

### 3.3 Correlation maps before and after data cleaning

**Fig. 6:**
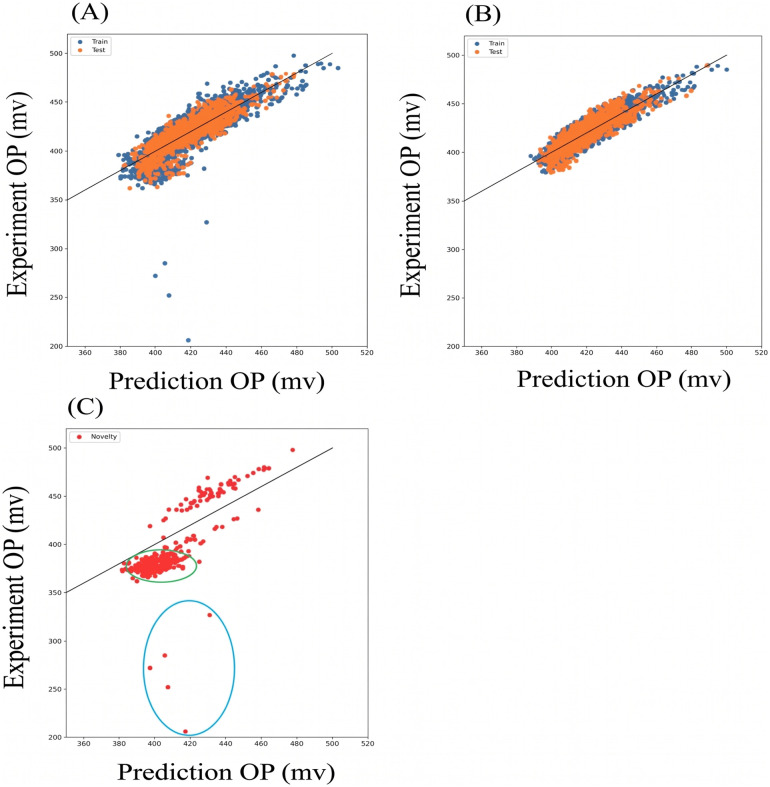
Correlation maps of predicted *versus* experimental OP values. (A) Entire dataset before cleaning. (B) Inlier set after cleaning, showing a strong linear correlation. (C) Outlier set with inliers removed, revealing a distinct cluster of low OP mode outliers.

The correlation maps of predicted *versus* experimental OP values before and after data cleaning highlight the impact of the cleaning process (Fig. 6). Prior to cleaning, the correlation map (Fig. 6A) exhibited significant scatter, indicating poor predictive performance. After cleaning, the inlier set (Fig. 6B) displayed a strong linear correlation with minimal dispersion, demonstrating the improved accuracy of the model. The outlier set (Fig. 6C), corresponding to the remainder of Fig. 6A subtracting Fig. 6B, revealed a distinct cluster of low OP mode outliers (circled in green), suggesting the emergence of a novel catalytic feature. This feature was obscured in the unprocessed dataset, underscoring the importance of isolating and analyzing outliers.^13^

### 3.4 Analysis of the outlier feature

**Fig. 7:**
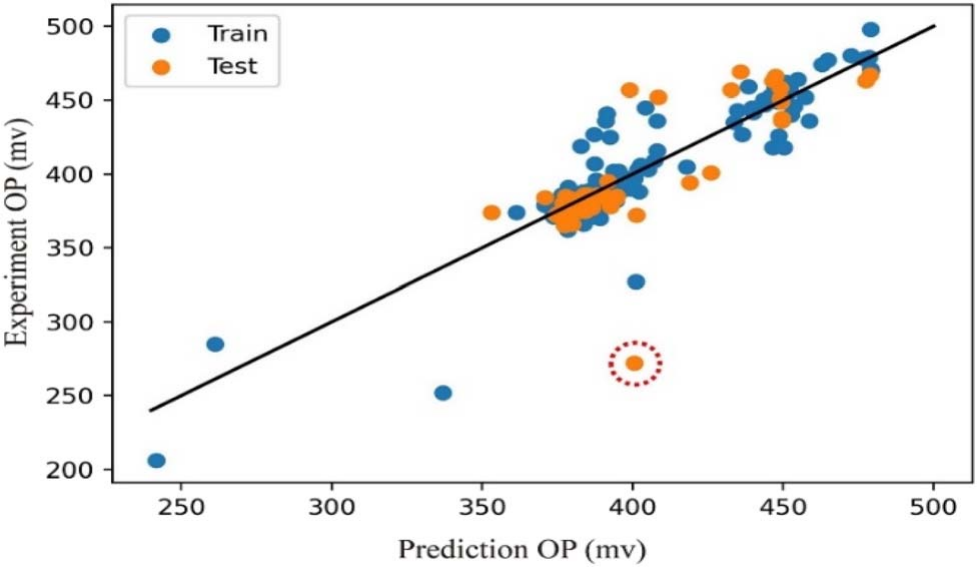
OP correlation map of the outlier set. The red circle marks an erroneous data point that disrupted model performance, corresponding to the point identified with arrow in Fig. 1 The remaining outliers display a distinct correlation pattern.

**Fig. 8:**
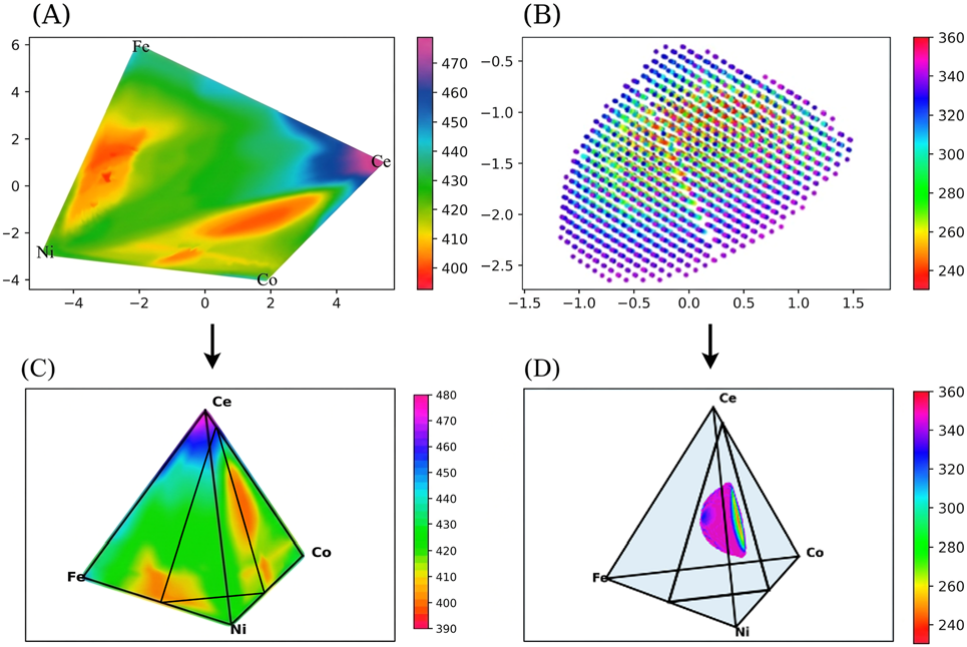
KPCA-projected overpotential (OP) distribution maps and corresponding tetrahedral coordinate transformations. (A) Inlier map showing a smooth and continuous OP gradient (420–460 mV), consistent with the established behavior of (Ni–Fe–Co–Ce)O_*x*_ catalysts. (B) Outlier map revealing discrete low-OP clusters (<360 mV) in a Ce-rich regime (0.3–0.6 at%), corresponding to high-activity domains. (C) Inlier data transformed into the experimental tetrahedral coordinate system, showing strong overlap with the empirical OP landscape. (D) Outlier data transformed into the same coordinate system, where low-OP clusters concentrate near the internal triangular plane (Co_0.1_Ce_0.9_–Ni_0.5_Fe_0.5_–Ni_0.5_Co_0.5_), consistent with experimentally observed high-performance regions.

The OP correlation map of the outlier set (Fig. 7) exhibits a distinct correlation pattern. For instance, a red circle marks an erroneous data point that disrupted model performance during training. Inclusion of this point in the training set prevented model convergence, whereas its removal improved the *R*^2^ test score from 0.62 to 0.80. While this improvement is significant, it must be acknowledged that, from a mathematical perspective, iteratively trimming data points with large prediction deviations will inevitably lead to an artificial increase in the *R*^2^ score. However, the critical validation of our approach lies in the non-random distribution of these removed points. If these outliers were merely random instrumental noise or measurement errors, they would be stochastically scattered across the entire compositional space. Instead, as subsequently illustrated in the KPCA outlier map (Fig. 8B) and the detailed compositional analysis (SI Table S3), these ∼330 anomalous data points are highly clustered within a specific Ce-rich regime (approximately 0.40–0.44 at%). This pronounced physical and chemical clustering firmly demonstrates that our anomaly detection framework is not simply stripping away random statistical noise; rather, it is successfully isolating a true chemical singularity that represents a novel, high-potential catalytic feature.

It is important to acknowledge that, from a conventional machine learning perspective, the iterative removal of high-deviation data points inevitably causes an artificial inflation of the *R*^2^ score, and the resulting value of 0.80 should not be interpreted as evidence of strong generalization capability across the full compositional space. The central purpose of this framework is not to construct a globally accurate regression predictor, but rather to employ prediction deviation as a diagnostic tool for uncovering hidden feature subspaces. In this context, the model functions as an anomaly-detection instrument rather than a conventional regression model, and cross-validation metrics designed for regression benchmarking are not the appropriate figure of merit. The most compelling validation of our approach is therefore not the *R*^2^ value itself, but the non-random, physically coherent clustering of the removed data points within the Ce-rich compositional regime an outcome that would be statistically implausible if the removed points represented random instrumental noise.

To rigorously validate the necessity of this framework, we benchmarked our NN + KPCA residual-based criterion against established unsupervised anomaly detection algorithms, including Isolation Forest (IF) and Local Outlier Factor (LOF). As detailed in SI Fig. S3 and S4, these traditional baseline methods failed to isolate the catalytically meaningful Ce-rich subset, confirming that standard density- or distance-based clustering cannot replace the proposed VisOut pipeline.

### 3.5 Exploration of OP distributions in inlier and outlier domains

The primary objective of this study is to identify composition activity motifs associated with low overpotential (OP) in multicomponent oxide catalysts. To this end, atomic descriptor–augmented features were employed to construct a 60-dimensional input space, enabling a more accurate and physically meaningful representation of structure–property relationships. Based on predictive deviation, the dataset was partitioned into inlier and outlier subsets, each subsequently visualized using Kernel Principal Component Analysis (KPCA). The resulting maps (Fig. 8) reveal two fundamentally distinct OP landscapes.

Fig. 8A, derived from interpolated predictions of the inlier subset, exhibits a smooth and continuous OP gradient (typically 400–470 mV), consistent with the well-established behavior of the (Ni–Fe–Co–Ce)O_*x*_ catalyst family. This map closely mirrors experimental references and reflects conventional composition–performance relationships typically captured by interpolation-based models.^29^ In contrast, Fig. 8B, representing the outlier domain, displays a fragmented and discontinuous topology characterized by isolated pockets of ultra-low OP (<360 mV). Compositional backtracking revealed that these low-OP clusters are concentrated in a Ce-enriched regime (0.3–0.6 at% Ce), consistent with previous reports of Ce-promoted oxygen evolution reaction (OER) activity.^30^

This clear divergence between inlier and outlier landscapes highlights two distinct forms of catalytic opportunity space. The inlier domain captures statistically dominant and predictable performance trends, while the outlier domain exposes sparsely populated, statistically marginal regions that exhibit unexpected catalytic efficiency. These regions, often overlooked by conventional interpolation frameworks, emerge only when descriptor-enriched representations and predictive deviation are used to explicitly treat outliers as analyzable entities.

To establish a direct link between the KPCA-derived features and experimental composition maps, both datasets were subsequently transformed into a tetrahedral coordinate system consistent with the experimental framework (Fig. 8C and D). Each vertex of the tetrahedron corresponds to Fe, Co, Ni, and Ce, with an internal triangular cross-section defined by the planes Co_0_._1_Ce_0_._9_–Ni_0_._5_Fe_0_._5_–Ni_0_._5_Co_0_._5_. This transformation aligns the latent KPCA projections with the physical compositional coordinates, enabling quantitative comparison with experimental trends rather than remaining in abstract feature space.

As shown in Fig. 8C, the inlier dataset exhibits a distribution that closely overlaps with the experimentally measured OP landscape, validating the model's fidelity in capturing empirical composition–activity relationships. In contrast, Fig. 8D shows that data points with OP < 360 mV are concentrated near the inserted triangular plane, forming a distinct low-OP region that coincides with the experimentally observed high-performance catalytic zone. This transformation bridges data-driven representations with physical interpretability, confirming that the apparent anomalies in the outlier domain correspond to compositionally coherent catalytic motifs.

Overall, these findings demonstrate that outliers in high-throughput materials datasets are not erroneous deviations but chemically informative indicators of emergent behavior. By integrating predictive deviation analysis, nonlinear dimensionality reduction, and coordinate transformation, this framework redefines outlier analysis from exclusion to exploitation offering a data-driven pathway toward the discovery of unconventional, high-efficiency catalysts.^18^

### 3.6 Ce composition trends within the outlier domain

To refine our understanding of the compositional origins of low OP values within the outlier dataset, the OP range was segmented into two intervals: a moderate-low OP zone (360–310 mV) and an ultra-low OP regime (310–235 mV), as summarized in SI Table S3. Analysis of Ce composition distributions revealed a clear shift in catalytic behavior across these boundaries. In the 360–310 mV range, data points were concentrated within the 0.35–0.44 at% Ce window, peaking at 0.40–0.44 at% with more than 500 unique compositions. As OP values decreased further into the 310–235 mV regime, the compositional peak shifted toward higher Ce content, specifically 0.40–0.49 at%, with the highest frequency observed in the 0.40–0.44 at% bracket (716 data points). This compositional transition suggests that slightly higher Ce incorporation facilitates a further reduction in OP.

These trends not only reinforce the earlier finding that Ce-rich domains (0.3–0.6 at%) are critical for achieving low OP values, but also narrow the most catalytically effective Ce window to approximately 0.40–0.44 at%. This range emerges as the most statistically robust and catalytically relevant zone in the outlier dataset, characterized by both a high density of data points and a strong correlation with ultra-low OP values. Notably, this refined insight aligns with previous *operando* and high-throughput studies, which likewise identified Ce doping as particularly effective in promoting OER activity.^30,31^

### 3.7 Data-driven mechanistic interpretation of Ce-rich catalysts

These findings indicate that the low OP values observed in the outlier set represent a distinct catalytic feature rather than experimental anomalies. The mechanisms discussed above not only corroborate the experimental observations but also motivate theoretical investigations of Ce-rich compositions. Future research should integrate density functional theory (DFT) simulations with experimental validation to elucidate the precise role of Ce in enhancing OER performance. In addition, generative models such as MatterGen may facilitate the design and analysis of innovative inorganic materials, thereby expanding the compositional space available for catalyst development.^32,33^

The pronounced reduction in overpotential (OP) observed for Ce-rich compositions within the outlier dataset can be attributed to a reproducible and compositionally distinct regime specifically, the 0.40–0.44 at% Ce window. Although Ce incorporation is generally known to promote charge transfer and oxygen evolution activity, our dataset reveals that even compositions with nearly identical global stoichiometry can display markedly different overpotentials. Within the Ce-rich region (≈0.40–0.44 at%), some samples fall into an exceptionally low-OP regime, while others remain in the typical range of 400–480 mV. This bimodality suggests that Ce's catalytic influence is not dictated by its bulk concentration, but rather by its spatial distribution and local electronic topology within the oxide lattice. We propose that low-OP samples correspond to microstructures where Ce forms surface or near-surface CeO_2−*x*_ nanoclusters, or Ce-enriched interfacial ribbons, embedded in the Ni–Fe–Co oxide matrix. These localized motifs act as redox buffers (Ce^4+^/Ce^3+^) and generate oxygen-vacancy-rich regions that facilitate OH/O formation and proton-coupled electron transfer. In this configuration, Ce enhances activity not by its abundance but by its ability to couple 4f orbitals with the 3d bands of neighboring transition metals, enabling percolative charge transport across the catalytic surface. Conversely, when Ce is too dispersed, buried in the bulk, or aggregated into large inactive domains, this interfacial coupling is lost, resulting in higher overpotentials.

The scarcity of low-OP samples further supports this structural interpretation. Forming the correct Ce topology requiring partial reduction to Ce^3+^, oxygen deficiency, and interfacial strain stabilization is energetically demanding and statistically rare under typical synthesis and annealing conditions. Thus, only a small fraction of samples achieve the necessary Ce clustering to lower the OER barrier, producing the minority low-OP population observed in the KPCA outlier domain. This explains both the reproducibility and the rarity of the phenomenon: low-OP behavior emerges only when Ce assumes specific interfacial configurations that are thermodynamically disfavored yet kinetically accessible in limited cases.

In this study, elemental compositions were supplemented with 14 chemically descriptive features such as NumValenceElectrons, NumRadicalElectrons, LabuteASA, and PEOE_VSA8 to enhance the model's ability to capture underlying physicochemical trends.^34–37^ These descriptors represent key properties related to electron availability, oxidation-state flexibility, surface accessibility, and charge distribution, all of which are mechanistically relevant to catalytic performance. During training, these features played a critical role in enabling the model to distinguish between inlier and outlier datasets, indicating that the predictive system implicitly learned signals associated with catalytic anomalies.^27–30^

We emphasize, however, that the role of these descriptors is fundamentally different from that of physics-derived descriptors such as the d-band center or DFT-calculated formation energies. The molecular descriptors employed here do not encode deep quantum-chemical causality, and we make no claim that the model itself deduced the microscopic mechanisms responsible for the observed low-overpotential behavior. Rather, the model served exclusively as a data-driven detector: its function was to objectively delineate the high-activity compositional window (0.40–0.44 at% Ce) from the surrounding inlier space. The mechanistic interpretation of why this window exhibits enhanced OER activity invoking Ce^3+^/Ce^4+^ redox buffering, oxygen-vacancy formation, and Ce 4f–Ni/Co 3d orbital coupling is a hypothesis constructed by domain experts drawing on existing experimental and theoretical literature,^29–31^ and is not a conclusion derived from the model output. This distinction between data-driven anomaly detection and expert-driven mechanistic hypothesis is a deliberate and essential feature of the proposed framework.

While no explicit causal link between Ce ratio and vacancy formation is encoded in the model, this data-driven correlation is supported by experimental studies demonstrating that Ce can modulate the oxidation states of Ni and Co *via* 4f–3d or 4f–2p–3d orbital interactions. Such interactions stabilize reactive oxygen intermediates and facilitate redox transitions throughout the catalytic cycle.^38–40^ Thus, the integration of chemical descriptors into the learning process not only enhances model performance but also provides interpretable connections to established mechanistic pathways in OER catalysis.

### 3.8 Research limitations and future prospects

Building upon the mechanistic insights discussed previously, the catalytic performance enhancement observed in the Ce-rich regime suggests that other 4f lanthanide elements with comparable electronic configurations might also offer potential pathways for catalyst design. However, while our deep learning-driven anomaly detection framework successfully pinpointed the optimal Ce-rich compositions without human intervention, we must acknowledge certain limitations in the current approach.

The input features employed in this study elemental compositions augmented with standard cheminformatics descriptors derived from RDKit are deliberately chosen for their computational accessibility and broad availability across compositional databases. We acknowledge that these descriptors do not encode deep quantum-chemical interactions such as orbital hybridization energies or d-band centers, and therefore cannot serve as a basis for inferring atomic-scale mechanisms. Crucially, however, this limitation does not undermine the validity of our core finding. The model's role in this framework is strictly that of a data-driven anomaly detector: it identifies which compositions deviate from the majority behavior, without needing to explain why. The subsequent mechanistic interpretation invoking Ce 4f–3d orbital coupling, oxygen-vacancy generation, and Ce^3+^/Ce^4+^ redox buffering is an expert hypothesis grounded in existing literature, not a model-derived conclusion. To further bridge the gap between anomaly detection and physical interpretability, future work will incorporate physics-informed feature representations including the Magpie elemental descriptor framework and DFT-calculated electronic descriptors such as the d-band center. Combining these richer representations with the VisOut™ anomaly detection pipeline and generative design tools such as MatterGen^18^ will establish a fully integrated and physically transparent pathway for next-generation electrocatalyst discovery.

## 4 Conclusions

In conclusion, this study serves as a compelling proof of concept (PoC) demonstrating the effectiveness of the VisOut deep learning framework in analyzing massive high-throughput experimental data. By intentionally isolating and analyzing outliers rather than discarding them, our approach successfully and autonomously identified a hidden, highly active catalytic feature associated with Ce-rich compositions (0.3–0.6 at%). The results confirm that these exceptionally low OP values are not experimental anomalies, but rather constitute a distinct compositional space with significant potential for enhancing OER performance. Importantly, the proposed framework also possesses the capacity to flag genuinely erroneous measurements, ensuring that the analysis does not indiscriminately treat all outliers as chemically meaningful.

Echoing our initial motivation, the successful “rediscovery” of this Ce-rich regime powerfully validates the robustness of our data-driven approach. The true potential of the VisOut framework, therefore, extends far beyond the current (Ni–Fe–Co–Ce)O_*x*_ system. When confronting completely unknown catalyst systems in the future, this automated pipeline can independently pinpoint emerging chemical singularities and automatically guide research and development directions. By significantly reducing the reliance on *a priori* chemical intuition and human trial-and-error, VisOut establishes a highly scalable, objective, and robust methodology for accelerating the discovery and optimization of next-generation materials.

## Author contributions

Chih-Yang Cheng: conceptualization, methodology, software, formal analysis, validation, visualization, writing – original draft. Yi-Huan Wu: data curation, validation, investigation, writing – review & editing. Feng-Yin Li: supervision, conceptualization, writing – review & editing.

## Conflicts of interest

There are no conflicts to declare.

## Supplementary Material

RA-016-D6RA02168A-s001

## Data Availability

All supplementary information (SI) for this study is provided in the SI. Supplementary information: list of 14 molecular descriptors used per element (Ni, Fe, Co, Ce). (SI Table S1). See DOI: https://doi.org/10.1039/d6ra02168a.
