# Supplementary material for: Deep learning-driven anomaly detection and feature discovery in Ce-rich (Ni–Fe–Co–Ce)Ox catalysts for oxygen evolution reaction
Source: RSC Adv. 2026 May 20;16(30):27117–25. doi: 10.1039/d6ra02168a (PMC13191585; doi:10.1039/d6ra02168a)
Supplement: RA-016-D6RA02168A-s001 [file RA-016-D6RA02168A-s001.pdf]

Supplementary Information (SI) for RSC Advances.
This journal is © The Royal Society of Chemistry 2026

Deep Learning-Driven Anomaly Detection and Feature Discovery in Ce-Rich (Ni-Fe-Co-Ce)Ox Catalysts for Oxygen Evolution Reaction

Chih-Yang Cheng[a], Yi-Huan Wu[b*], and Feng-Yin Li[a*]

[a] Department of Chemistry, National Chung Hsing University, Taichung 402, Taiwan. E-mail: feng64@nchu.edu.tw
[b] Department of Chemistry, R. O. C. Military Academy, Kaohsiung, Taiwan. E-mail: m1090008@rocma.edu.tw

RSC Advances

Supplementary Information

Table S1 List of 14 Molecular Descriptors Used per Element (Ni, Fe, Co, Ce). Descriptors employed in the OP predictor model for feature engineering of overpotential (OP) values. Each element (Ni, Fe, Co, Ce) was represented in SMILES format (e.g., [Ni]) and evaluated using the RDKit MolecularDescriptorCalculator. The 14 descriptors listed in Table S1 were computed independently for each element, resulting in a total of 14 × 4 = 56 descriptors. Together with the four elemental composition ratios (Ni, Fe, Co, Ce), these descriptors constituted the complete set of 60 input features used for model training and prediction (see Section OP predictor: Feature Engineering of OP values in the main text).

Descriptor	Ni	Fe	Co	Ce	Description
qed	0.3441	0.3423	0.3680	0.4440	Quantitative Estimate of Drug-likeness; originally for drug-like molecules, used here as a general molecular descriptor.
ExactMolWt	57.9353	55.9349	58.9331	139.9054	Exact molecular weight based on isotopic composition.
NumValenceElectrons	10	8	9	4	Number of valence electrons in the atom/molecule.
NumRadicalElectrons	0	0	1	0	Number of radical (unpaired) electrons.
Chi0n	0.3162	0.3535	0.3333	0.5	Kier–Hall connectivity index (order 0, non-valence form), reflecting atomic topology.
Chi0v	1.3038	1.4577	1.3743	3.6400	Kier–Hall connectivity index (order 0, valence form), incorporating valence state information.
HallKierAlpha	0.4935	0.5194	0.5064	1.3766	Kier's alpha parameter, an index for molecular branching and flexibility.
Kappa1	1.4935	1.5194	1.5064	2.3766	First-order molecular shape index (reflecting molecular size and branching).
Kappa2	0.5198	0.4444	0.4808	0.1030	Second-order molecular shape index.
Kappa3	4.5987	4.2194	4.4039	0.2822	Third-order molecular shape index.
LabuteASA	17.4152	17.9640	17.6885	41.2512	Approximate solvent accessible surface area (ASA).
PEOE_VSA8	16.4914	17.0686	16.7787	41.7472	Partial Equalization of Orbital Electronegativities-based surface area (bin 8).
EState_VSA2	16.4914	17.0686	16.7787	41.7472	Electrotopological State (E-State) index partitioned by van der Waals surface area (bin 2).
MolLogP	-0.0025	-0.0025	-0.0025	0	Octanol-water partition coefficient (logP), measuring hydrophobicity.

Table S2 Hyperparameters and Architecture for the Deep Learning Regression Model. This table details the network architecture and training hyperparameters used for the overpotential (OP) predictor

Hyperparameter / Setting	Value / Description
Input Features	60 (4 elemental compositions + 56 molecular descriptors)
Hidden Layers Configuration	3 fully connected layers (256, 128, and 64 neurons)
Activation Function	Leaky ReLU (slope = 0.2)
Dropout Rate	0.25 (to mitigate overfitting)
Optimizer	Adam
Learning Rate	0.001
Batch Size	32
Epochs	250
Loss Function / Metric	Mean Absolute Error (MAE) / Mean Squared Error (MSE)
Train/Test Split Ratio	8:2

Table S3 The distribution of Ce composition ratios in the outlier set with the OP values around the boundary of the inliers (360 ~ 310 mV) and in the new low region (310 ~ 235 mV).

360 ~ 310 mV		310 ~ 235 mV	
Ce composition at%	# of data points*	Ce composition at%	# of data points*
0.20 ~ 0.24	0	0.20 ~ 0.24	0
0.25 ~ 0.29	135	0.25 ~ 0.29	0
0.30 ~ 0.34	424	0.30 ~ 0.34	341
0.35 ~ 0.39	490	0.35 ~ 0.39	660
0.40 ~ 0.44	510	0.40 ~ 0.44	716
0.45 ~ 0.49	421	0.45 ~ 0.49	593
0.50 ~ 0.54	358	0.50 ~ 0.54	322
0.55 ~ 0.59	167	0.55 ~ 0.59	73
0.60 ~ 0.64	0	0.60 ~ 0.64	0

* The four metal composition ratios of the data points, together with their corresponding OP values, are provided in the Supplementary Materials. These data points were selected from 152,748 discrete compositional combinations.

Please do not adjust margins

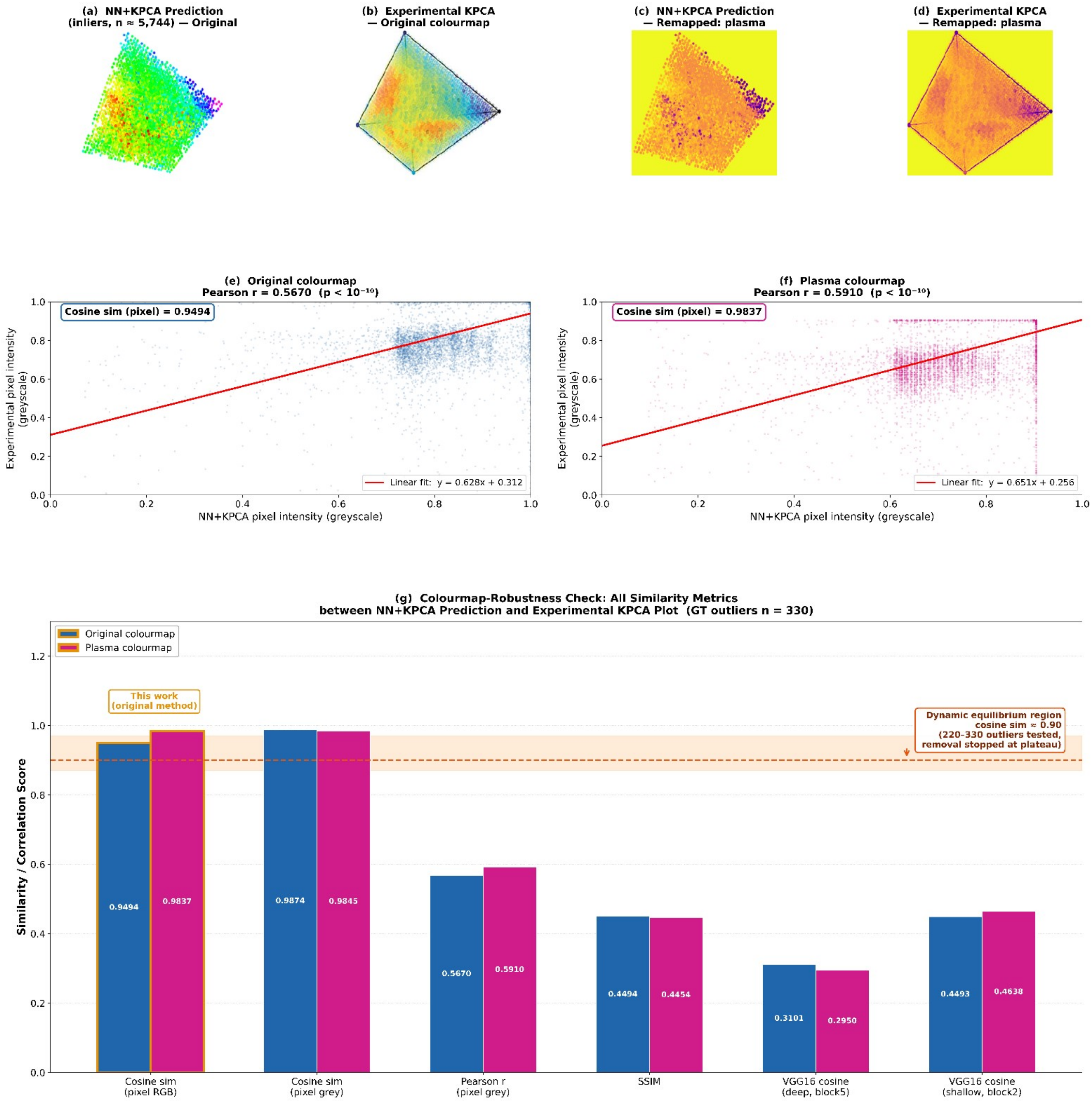

Fig. S1 Empirical validation of the pixel-vector cosine similarity metric used to assess convergence between the NN+KPCA prediction and the experimental KPCA plot. (a) KPCA scatter plot of the neural-network-predicted overpotential for the retained inlier dataset (n ≈ 5,744 samples; original colourmap). (b) KPCA scatter plot of the corresponding experimental overpotential values (original colourmap). (c, d) Same plots as (a) and (b) remapped to the perceptually uniform plasma colourmap to assess colourmap dependence. (e, f) Pixel-level Pearson correlation between the greyscale representations of (a) vs. (b) and (c) vs. (d), respectively. Each point represents one pixel; the red line is the linear fit. Both colourmaps yield statistically significant positive correlations ($r > 0.56$, $p < 10^{-10}$), confirming genuine spatial correspondence between the two KPCA surfaces. (g) Summary of all six similarity metrics computed under the original and plasma colourmaps. The cosine similarity formula $\cos(A, B) = (A \cdot B)/(\|A\| \cdot \|B\|)$, where A and B are flattened RGB pixel vectors, is the metric used in this work (gold border). The orange shaded band (0.87–0.97) and dashed line (0.90) indicate the dynamic equilibrium region: during iterative outlier removal (220–330 candidates tested), the cosine similarity stabilised near 0.9 with no further increase, at which point removal was stopped. Metric values are consistent across both colourmaps (deviation < 2%), confirming that the measured convergence reflects genuine data agreement rather than a colourmap artefact. VGG16 deep-feature cosine similarity values (0.31–0.46) are lower by design, as they encode semantic texture distances rather than direct spatial correspondence.

Please do not adjust margins

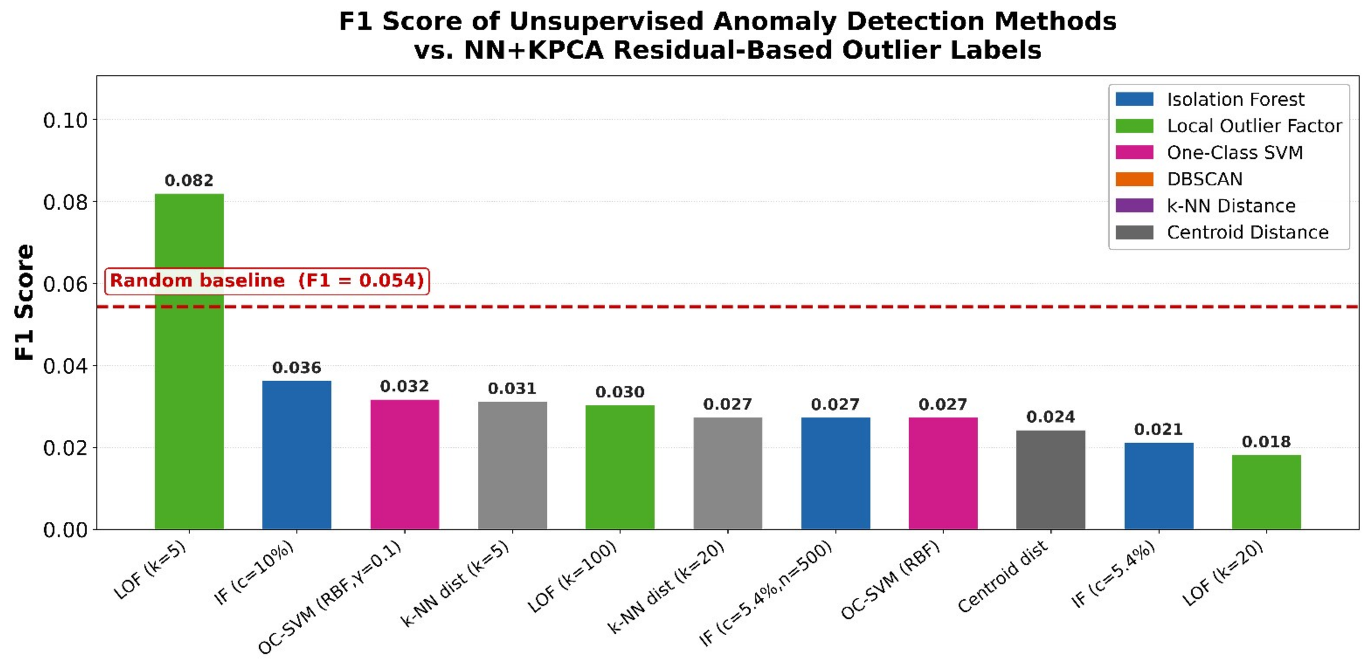

Fig. S2 F1 score comparison of unsupervised anomaly detection methods against NN+KPCA residual-based outlier labels. Bar chart showing the F1 scores of thirteen unsupervised anomaly detection configurations spanning six algorithm families: Isolation Forest (IF; blue), Local Outlier Factor (LOF; green), One-Class Support Vector Machine (OC-SVM; pink), DBSCAN (orange), k-nearest-neighbour distance (k-NN dist; purple), and centroid distance (grey). The red dashed line indicates the expected F1 score of a random classifier at the observed outlier prevalence (5.4%; F1 = 0.054). All methods yield F1 scores at or below this baseline, demonstrating that geometric anomalies in the 60-dimensional chemical descriptor space do not correspond to the 330 outliers identified by the neural-network + kernel principal component analysis (NN+KPCA) residual criterion.

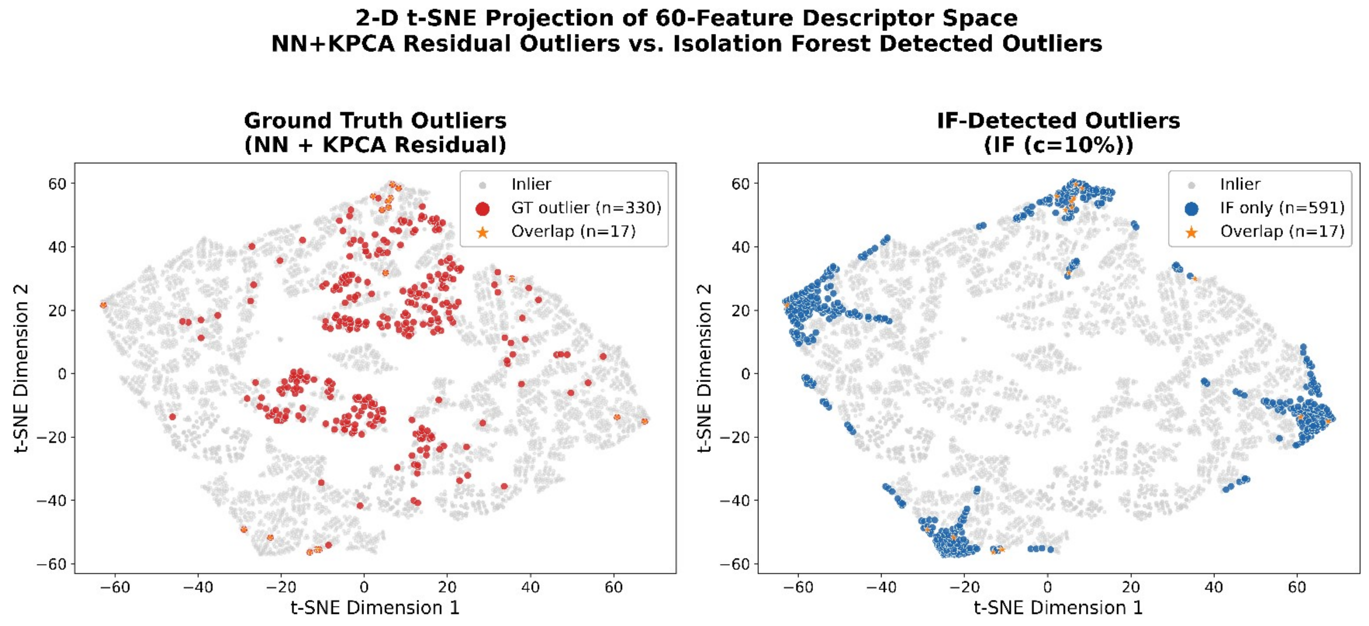

Fig. S3 Two-dimensional t-SNE projection of the 60-feature descriptor space comparing ground-truth and Isolation Forest-detected outliers. Each point represents one of the 6,074 samples, projected from 60 dimensions to two dimensions using t-distributed stochastic neighbour embedding (t-SNE; perplexity = 40). (Left) Ground-truth outliers (red; n = 330) identified by the NN+KPCA residual criterion are distributed throughout the entire feature space without spatial clustering. (Right) Outliers detected by Isolation Forest (blue; c = 10%) are concentrated at the compositional boundaries of the feature space. Orange stars indicate the rare overlap between the two sets (n = 17), confirming that the two outlier definitions are nearly orthogonal and capture fundamentally different sample characteristics.

This journal is © The Royal Society of Chemistry 20xx

Please do not adjust margins

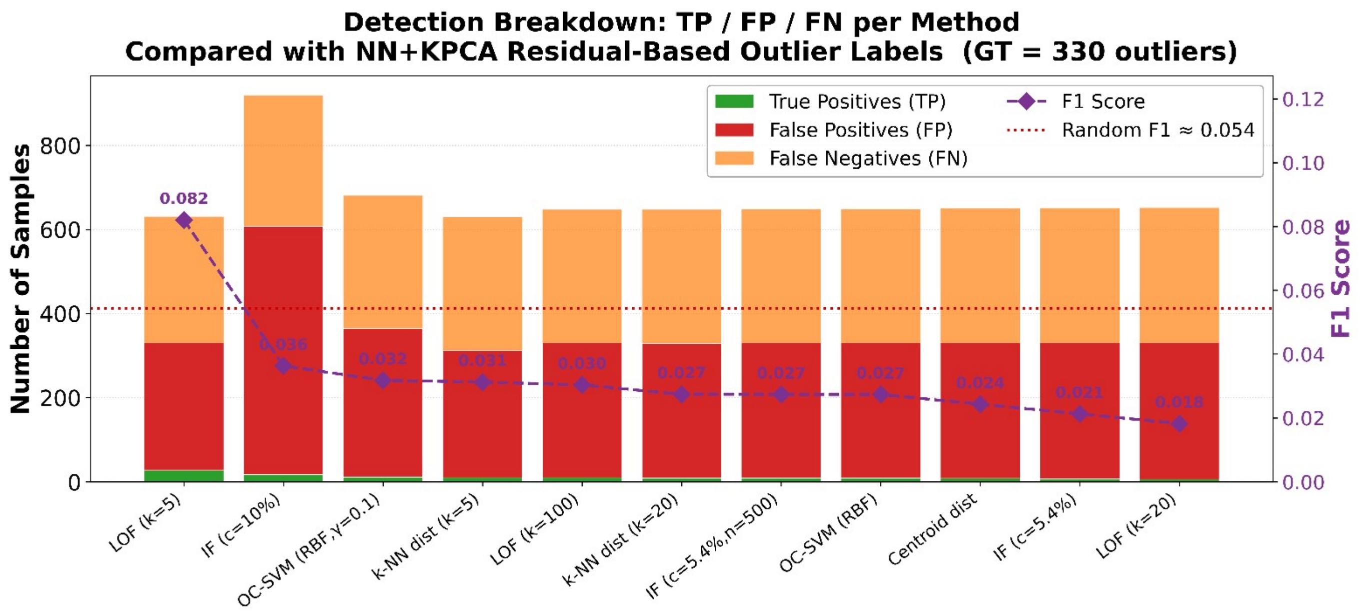

Fig. S4 Per-method detection breakdown showing true positives (TP), false positives (FP), and false negatives (FN) relative to the NN+KPCA residual-based outlier labels (n = 330). Stacked bars (left axis) quantify, for each method, the number of correctly recovered outliers (TP; green), incorrectly flagged inliers (FP; red), and missed outliers (FN; orange). The superimposed dashed line with diamond markers (right axis, purple) shows the corresponding F1 score for each method; the red dotted line marks the random-classifier F1 baseline (≈ 0.054). Across all configurations, TP counts remain negligible relative to FP and FN, and no method achieves an F1 score substantially above the random baseline, further corroborating that unsupervised feature-space anomaly detection cannot serve as a surrogate for the NN+KPCA residual-based outlier identification strategy.

Please do not adjust margins
